# Selective Production of Phenol on Bifunctional, Hierarchical ZSM-5 Zeolites

**DOI:** 10.3390/molecules26123576

**Published:** 2021-06-11

**Authors:** Margarita Popova, Ágnes Szegedi, Manuela Oykova, Hristina Lazarova, Neli Koseva, Magdolna R. Mihályi, Pavletta Shestakova

**Affiliations:** 1 Institute of Organic Chemistry with Centre of Phytochemistry, Bulgarian Academy of Sciences, 1113 Sofia, Bulgaria; Manuela.Oykova@orgchm.bas.bg (M.O.); Hristina.Lazarova@orgchm.bas.bg (H.L.); Pavletta.Shestakova@orgchm.bas.bg (P.S.); 2 Research Centre for Natural Sciences, Institute of Materials and Environmental Chemistry, Magyar tudósok krt.2., 1117 Budapest, Hungary; mihalyi.magdolna@ttk.hu; 3 Institute of Polymers, Bulgarian Academy of Sciences, 1113 Sofia, Bulgaria; koseva@polymer.bas.bg

**Keywords:** hierarchical ZSM-5 zeolite, bifunctional catalysts, phenol production, gas phase reaction

## Abstract

Mono- and bimetallic Ni-, Ru- and Pt-modified hierarchical ZSM-5 materials were prepared by impregnation technique and characterized by X-ray diffraction (XRD), N_2_ physisorption, temperature-programmed reduction (TPR–TGA), ATR–FTIR and solid state NMR spectroscopy. Formation of finely dispersed nickel, ruthenium and platinum species was observed on the bimetallic catalysts. It was found that the peculiarity of the used zeolite structure and the modification procedure determine the type of formed metal oxides and their dispersion and reducibility. The samples’ acidity was studied via FTIR spectroscopy of adsorbed pyridine. The changes in the zeolite structure were studied via solid-state NMR spectroscopy. The catalysts were investigated in a gas-phase hydrodeoxygenation, transalkylation and dealkylation reaction of model lignin derivative molecules for phenol production.

## 1. Introduction

Significant development in industrial activities has caused environmental hazards, irreparable damage to ecosystems and adverse consequences for human health [1,2,3]. The replacement of fossil fuels with alternative energy sources could address this problem. Lignocellulosic biomass is a promising inexpensive renewable material that could satisfy society’s requirements for chemicals and fuels. Lignin, cellulose and hemicellulose are the three principal components of lignocellulosic biomass. Lignin is the main component of lignocellulosic biomass (15–30 wt%) and is still not sufficiently used in biorefineries [3,4,5,6]. The first step in its valorization is lignin depolymerisation via hydrogenolysis [7,8], oxidation [9], hydrolysis [10,11], etc. The depolymerized lignin is most often a complex mixture of phenolic compounds, hampering direct use as a high-value chemical. Subsequent catalytic processes, including selective demethoxylation and dealkylation, lead to the transformation of substituted phenols into phenol. Phenol is one of the most important aromatic chemicals used in industry with 8.9 million tons produced annually [12], which are mainly used as synthetic polymer precursors, pharmaceuticals and herbicides. Among others, ethyl- and propylphenols are the main aromatics derived from the lignin depolymerization process [13]. Catalytic conversion of alkylphenols in the gas phase to phenol and alkenes was recently reported over acidic catalysts (zeolites, ZrO_2_) [14,15]. Transalkylation is considered a promising new method for producing phenol from alkylphenols [16]. When toluene is employed as the recipient of the alkyl group, alkylated benzenes are co-produced, which can then be applied as additives to gasoline or jet fuels due to their high octane number and high calorific value. Many studies have focused on the decomposition of the methoxy group in phenols, i.e., demethoxylation, which is widely applied for producing biofuel from lignin [3,4]. Many catalysts have been studied in hydrodeoxygenation reactions, including supported noble and transition metals (Ru, Rh, Pd, Pt, Ni, Co, Fe) on different types of acidic supports (Al_2_O_3_, USY, MCM-22, BEA, ZSM-5, etc.) [17,18,19,20,21,22,23].

The improvement of accessibility to the acidic sites of zeolites is a promising approach and many synthesis methods have been developed. The preparation of zeolites with a hierarchical pore structure by direct synthesis and by post-synthesis treatments has been exhaustively investigated through the years by many researchers. Acidic treatment with a mixture of NH_4_F and HF leads to simultaneous removal of Si and Al, which is very important for preservation of structure and Si/Al ratio [23,24,25]. Zeolites modified by this method show greater accessibility to the active acidic sites, promoting higher catalytic activity, especially in the case of bulky and branched molecules, where the diffusion restrictions limit the reaction rate.

In this study, a hierarchical ZSM-5 zeolite was developed and its monocomponent and bimetallic modifications with Ni, Ru and Pt were investigated in the gas-phase hydrodemethoxylation, transalkylation and dealkylation of 2-methoxy-4-propylphenol and 4-ethylphenol.

## 2. Results and Discussion

### 2.1. Physico-Chemical Properties

**Figure 1:**
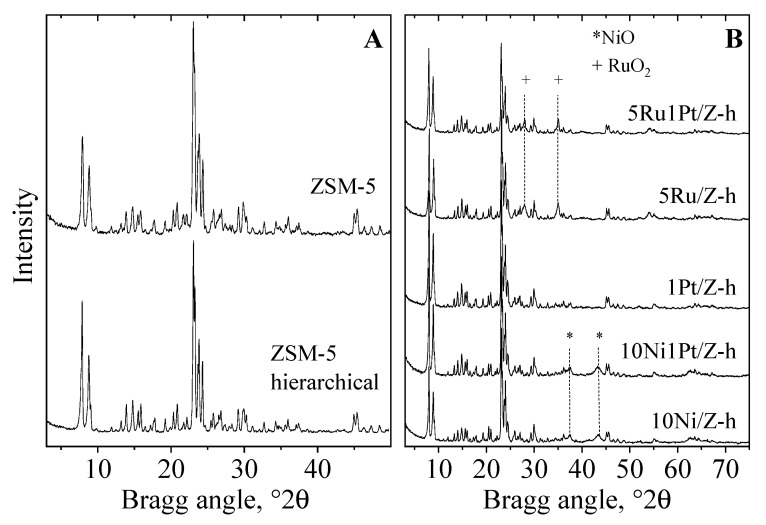
X-ray powder diffraction patterns of (**A**) initial and hierarchical ZSM-5 catalysts, and (**B**) the Ni-, Pt- and Ru-modified varieties.

Hierarchical ZSM-5 was prepared by treatment with a mixture of HF and NH_4_F. XRD patterns of parent and hierarchical ZSM-5 samples were identical (Figure 1A), and the crystalline structure of the zeolite was not damaged by treatment with HF and NH_4_F.

XRD data of the mono- and bimetallic nickel-, ruthenium- and platinum-containing zeolite materials also evidenced the preserved crystalline structure, and the presence of RuO_2_ and NiO nanoparticles [26] in the corresponding catalysts (Figure 1B). However, no traces of crystalline metallic platinum or platinum oxide compounds could be detected. It seems that Pt particles were either smaller than 5 nm [27] or occupied ion-exchange positions in the zeolite lattice. The crystallite size of nickel oxide particles as determined by the Scherrer equation with profile fitting method was below 10 nm (Table 1), whereas RuO_2_ nanoparticles were much bigger, 23–35 nm.

While Ni- and NiPt-containing samples (Table 1) showed similar metal-oxide dispersion, the ruthenium-modified ones were different. Bigger RuO_2_ particles were formed on the platinum-containing ruthenium catalyst by the applied synthesis method.

The metal content was determined by ICP analysis and the results confirmed that the whole amount of the impregnating metals could be found on the support (Table S1).

**Figure 2:**
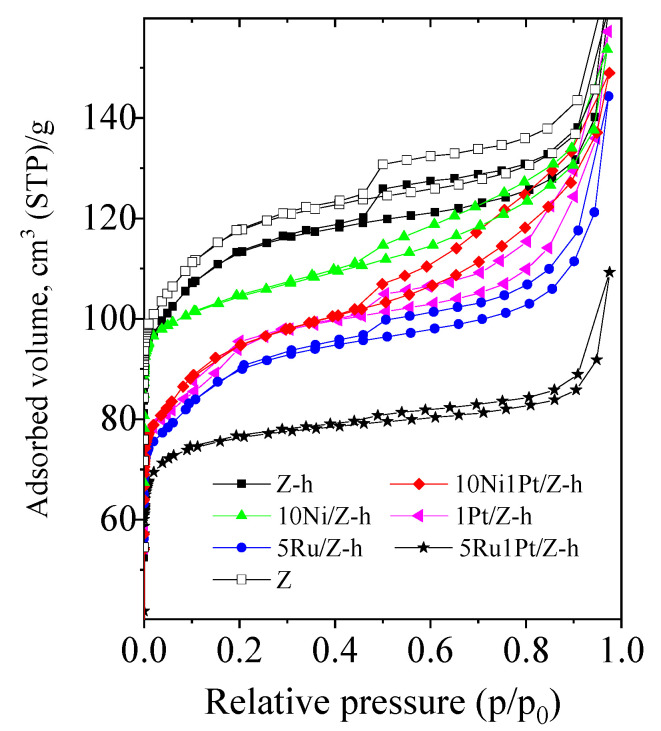
Nitrogen adsorption/desorption isotherms of Z-h and the Ni-, Ru- and Pt-modified Z-h zeolites.

In Figure 2, nitrogen physisorption isotherms of the initial, hierarchical ZSM-5 zeolite and metal-containing zeolite materials are presented. The corresponding calculated parameters are listed in Table 1. The correlation coefficients of the BET curves (R) were over 0.999 in all cases (Figure S1). The preparation of Z-h led to an increase in mesopore volume (Table 1), which is evidence for the formation of a hierarchical pore structure. The impregnation procedure resulted in some decrease in specific surface area of the modified hierarchical ZSM-5 zeolite (Table 1). This means that some pores were partly blocked by the metal/metal-oxide nanoparticles. A negligible decrease in the surface area was detected for 1Pt/Z-h, whereas it was more significant for the 5Ru1Pt/Z-h sample, probably also due to deposition of ruthenium-oxide nanoparticles in the mesopores.

**Figure 3:**
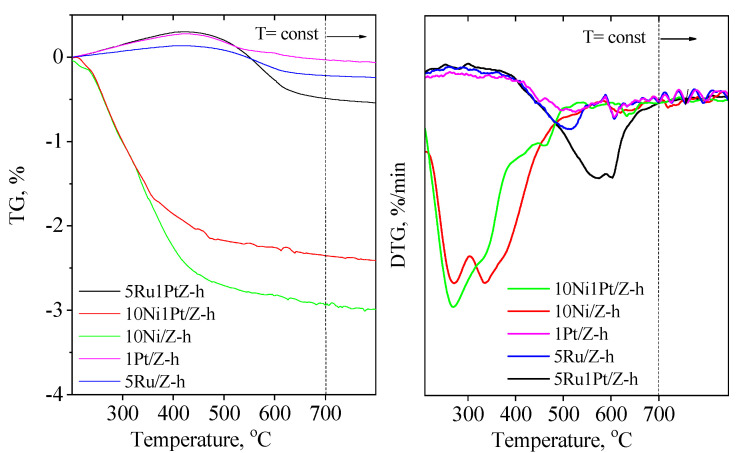
TG curves and TPR profiles of the Ni-, Ru- and Pt-modified hierarchical ZSM-5 zeolites.

TPR–TG profiles of the nickel, ruthenium and platinum-modified mono- and bimetallic materials are shown in Figure 3. The TPR curves of 5Ru/Z-h and 5Ru1Pt/Z-h samples showed reduction peaks of lower intensity at higher temperatures between 400 and 600 °C. The TPR curves of 10Ni/Z-h and 10Ni1Pt/Z-h samples were characterized by two overlapping peaks between 200 and 450 °C. The reduction step at lower temperatures can probably be related to the reduction in finely dispersed NiO particles. The higher temperature peak can be associated with the reduction in nickel ionic species incorporated into the zeolite lattice during the salt decomposition procedure by a solid-state ion-exchange procedure [28]. We infer that the presence of Ni strongly bound to the support positively influenced the platinum state and stabilized the dispersion of NiO particles.

**Figure 4:**
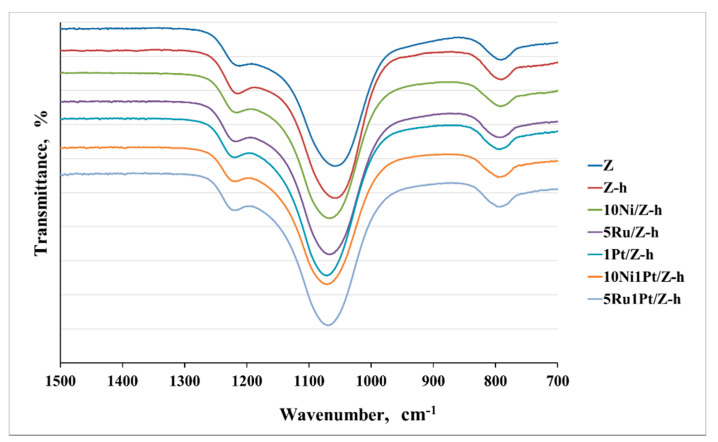
ATR-FTIR spectra of the studied samples.

ATR–FTIR spectra of the studied samples are presented in Figure 4. The broad and intensive peak at 1059 cm^−1^ for ZSM-5 and ZSM-5-h was attributed to the asymmetric stretching of SiO_4_ [29,30]. The shift of the peak at 1059 cm^−1^ to higher wavenumbers was due to the incorporation of metal cations into the zeolite structure or changes in the Al incorporation, which was due to the different Si/Al ratio after treatment with HF and NH_4_F [31]. The shift was more pronounced for the 10Ni1Pt-ZSM-5 catalyst and the peak was detected at 1072 cm^−1^. The stronger interaction of Ni and Pt with the support in the bimetallic catalyst was suggested by TPR experiments and could explain the observed changes in the FTIR spectrum. The shifts were smallest for 10Ni-ZSM-5-h and 5Ru-ZSM-5-h, in which the formation of crystalline metal oxides was detected.

**Figure 5:**
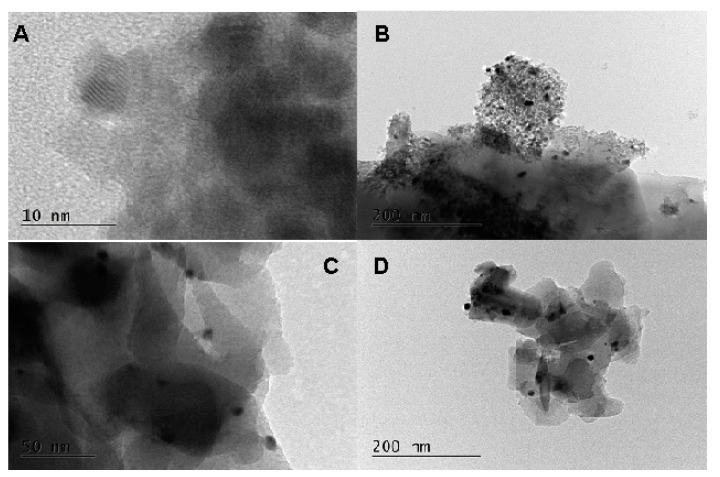
TEM images of 10Ni1Pt/Z-h (**A**,**B**) and 5Ru1Pt/Z-h (**C**,**D**) zeolites.

TEM images of the 10Ni1Pt/Z-h sample show the presence of NiO and Pt nanoparticles (Figure 5A,B). Formation of bigger metal particles was registered in the 5Ru1Pt/Z-h catalyst due to the presence of RuO_2_, as shown by XRD (Figure 5C,D). The presence of Pt and Ni particles in 10Ni1Pt/Z-h was proven by SAED (Figure S2).

#### 2.1.1. FTIR Spectra of Adsorbed Pyridine

**Figure 6:**
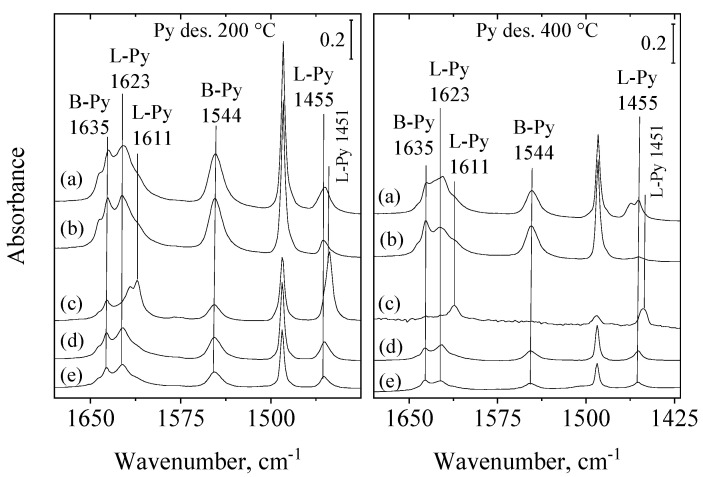
FTIR spectra of adsorbed pyridine on the studied catalysts: (**a**) parent ZSM-5, (**b**) hierarchical Z-h, (**c**) 10Ni/Z-h, (**d**) 1Pt/Z-h, (**e**) 5Ru/Z-h. Py (6 mbar) was adsorbed on 400 °C dehydrated samples at 200 °C, and desorbed at 200 and 400 °C in high vacuum.

The acidic properties of selected catalysts were characterized by FTIR spectroscopy of adsorbed pyridine (Py) in order to compare their Brønsted and Lewis acidity [32]. The FTIR spectra of the investigated samples are presented in Figure 6. The 8a, 8b and 19b ring vibrations (ν_CCN_) of Py yielded adsorption bands in the 1700–1400 cm^−1^ frequency region [33]. Py can be coordinated to Lewis acid sites (L-Py) or protonated on Brønsted acid centers (B-Py) of zeolites. B-Py showed a band pair at approximately 1545/1635 cm^−1^, whereas L-Py bands appeared at 1445–55 and 1610–23 cm^−1^ frequencies. In zeolites Brønsted acidity stems from tetrahedrally coordinated aluminum species, where the negative charge of the framework is compensated by a proton, i.e., from bridged hydroxyl groups ((≡Si–O)_3_≡Al–(OH)–Si≡). Lewis acid sites are identified as Py coordinated to coordinatively unsaturated lattice cations, such as extra-framework AlO^+^ species or other metallic/transition metallic ones. The desorption temperature of pyridine is characteristic for the strength of acidity. In zeolites Py could not be desorbed from the Brönsted and Lewis sites even at 400 °C, indicating its very strong acidic character.

Both Lewis and Brønsted type acid sites could be detected on the parent ZSM-5 zeolite (Figure 6a). The 1455/1623 cm^−1^ band pair could be associated with Py coordinatively bound to AlO^+^ species. The Brønsted acidity of hierarchical ZSM-5 was identical with that of starting ZSM-5 (Figure 6b); the amount of Lewis acid sites was decreased, probably due to removal of extra framework cations by HF in the form of AlF_3_. Thus, FTIR spectroscopic results also supported that the HF/NH_4_F treatment did not detectably affect the framework structure of the zeolite. By impregnating the hierarchical ZSM-5 with different metals, the Brønsted acidity of the catalysts significantly decreased (Figure 6c–e). In the case of the 10Ni-Z-h sample (Figure 6c) the appearance of a new, intense band at 1451/1611 cm^−1^ was evidence of the incorporation of Ni^2+^ ions into cationic positions. It seems that during the impregnation procedure, ion exchange also occurred, and the formed nitric acid could adversely affect the acidity of the catalyst. In the 400 °C desorption spectra, Py was totally desorbed from the latter sample, indicating decreased acid strength. Other catalysts in smaller amounts still showed B-Py ring vibration even at higher temperature desorption. No incorporation of Pt or Ru ions into zeolite lattice positions could be detected by FTIR spectra.

#### 2.1.2. NMR Analysis

The parent, hierarchical and Ru-, Pt- and Ni-modified ZSM-5 zeolites were investigated with ^29^Si and ^27^Al NMR spectroscopy. The ^29^Si NMR chemical shifts depend on the type and number of tetrahedrally coordinated T atoms (T = Si, Al) in the second coordination sphere of the SiO_4_ unit. The five different structural environments of the tetrahedrally coordinated Si atoms, Si(OSi)_4-n_(OT)_n_, designated as Si(nT), have characteristic chemical shifts in the ^29^Si NMR spectra, while the relative intensities of the signals for the different Si(nT) species reflect the composition of the zeolite framework (*n* = 0, 1, 2, 3 and 4 represents the number of Al atoms sharing oxygen with the SiO_4_ unit). In addition to the chemical environment, the ^29^Si chemical shifts are also sensitive to Si-O bond lengths and Si-O-T bond angles in the crystallographically inequivalent Si sites. The ^29^Si NMR spectra can be used to calculate the framework Si/Al ratio from the NMR signal areas (I) according to Equation (1) [34]:

(1) $$\mathrm{Si}/T=\frac{\sum_{n=0}^{4}I_{\mathrm{Si}\left(\mathrm{nT}\right)}}{\sum_{n}^{4}0.25{\mathrm{nI}}_{\mathrm{Si}\left(\mathrm{nT}\right)}}$$

**Figure 7:**
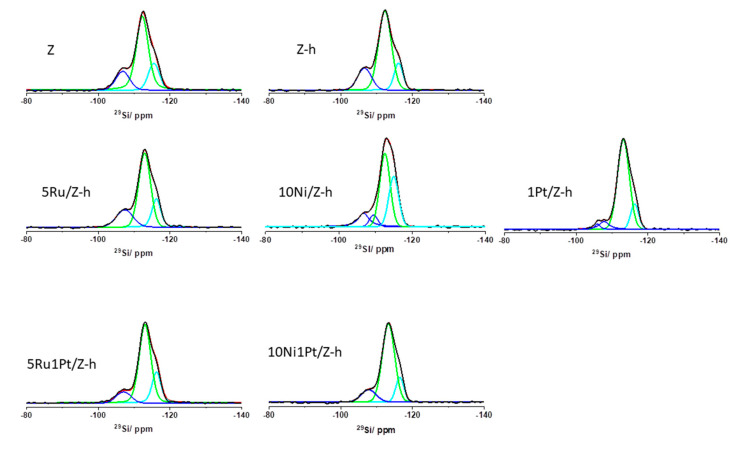
Experimental (black) and simulated (red) single-pulse ^29^Si NMR spectra of the studied zeolites. The individual contributions of the different Si environments are given with colored lines (green, blue and cyan).

The single-pulse ^29^Si NMR spectra of the studied zeolites demonstrated three main resonances with chemical shifts at approximately −107 ppm, −113 ppm and −115 ppm after fitting the spectral patterns with DMFit software [35], (Figure 7).

According to data from the literature, the intense resonance at approximately −113 ppm and the shoulder at −115 ppm are assigned to two inequivalent crystallographic tetrahedral Si(0Al) sites, while the signal at –107 ppm originates from Si(1Al) species [36,37]. The results for the Si/Al ratio in the studied mono- and bi-component modified ZSM5-h zeolites calculated using the areas of the fitted lines are summarized in Table 2.

The data presented in Table 2 show that the Si/Al ratio is preserved in the monometallic Ru- and Ni-modified zeolites (5Ru/Z-h and 10Ni/Z-h). Modification with Pt resulted in a higher Si/Al ratio, indicating partial dealumination of the zeolite framework as a result of the introduction of Pt. The highest degree of dealumination was detected in the bimetallic system modified with Ru and Pt (5Ru1Pt/Z-h, Si/Al = 40).

**Figure 8:**
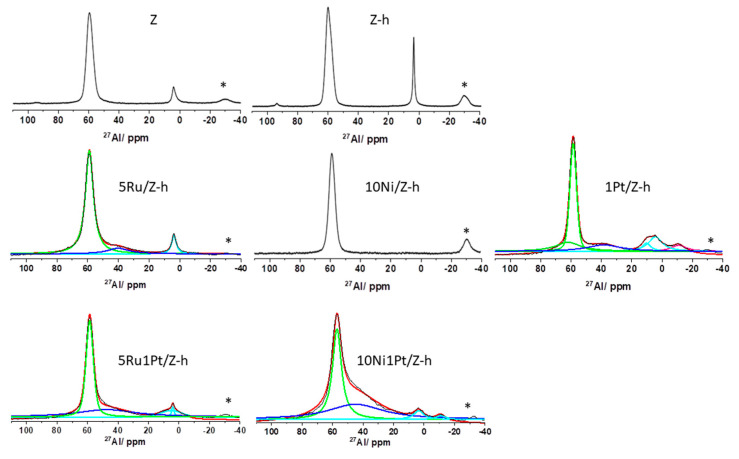
Experimental (black) and simulated (red) single-pulse ^27^Al NMR spectra of the studied zeolites. The individual contributions of the different Al environments are given with colored lines (green, blue and cian). The FAl:EFAl ratio for ZSM-5 and ZSM-h was determined by direct integration of the two resonances. (* denotes a spinning side band).

The ^27^Al NMR spectra gave information about the coordination state of Al nuclei and the symmetry of their structural environment in the zeolite framework. ^27^Al NMR spectra of the parent ZSM-5 and hierarchical Z-h zeolites (Figure 8) showed two resonances at −60 ppm and at approximately 0 ppm, characteristic of tetracoordinated framework Al sites (FAl) and of six-coordinated extra-framework Al species (EFAl), respectively. The FAl:EFAl ratio was 91:9 for the parent ZSM-5 zeolite and 71:18 for the hierarchical Z-h zeolite, indicating an increase in the amount of EFAl species as a result of the treatment. Modification with Ru, Ni and Pt resulted in different degrees of structural changes as a result of the introduction of metal atoms. These structural transformations were mainly associated with distortion of the environmental symmetry of some Al sites, demonstrated by the appearance of a broad resonance at approximately 40–38 ppm. [38,39,40]. The areas of the different ^27^Al resonances obtained by deconvolution of the ^27^Al spectra are presented in the last three columns of Table 2. The mono- and bi-component Pt-modified zeolites also showed the presence of at least two different types of EFAl species represented by a narrow resonance at approximately 4 ppm and a broad signal centered at approximately 7 ppm. Two of the Pt-modified zeolites, 1Pt/Z-h and 10Ni1Pt/Z-h, also showed a resonance at −11 ppm of unknown origin. The highest amount of the EFAl species was detected in the 1Pt/Z-h sample, while in the 10Ni/Z-h zeolite, only tetracoordinated framework Al sites were present.

The dealumination and structural changes in the zeolite framework are often associated with the formation of silanol groups. The chemical shift range of the Si(1OH) groups is approximately −102 ppm and therefore the signal partially overlaps with that of the Si(1Al) at –107 ppm. This leads to some uncertainty in the determination of the Si/Al ratio from the one-pulse ^29^Si spectra, since part of the intensity of the –102 ppm signal, originating from the SiOH groups, must be attributed to the Si(0Al) units rather than to the Si(1Al) units. To qualitatively identify the presence of SiOH structural units generated at defective sites in the zeolite framework of the studied materials, ^1^H→^29^Si CP–MAS spectroscopy was used. The cross polarization (CP) technique is based on transfer of polarization from abundant spins (^1^H) to low-sensitivity nuclei (^29^Si) via space dipole–dipolar interactions, resulting in selective enhancement of the signals from ^29^Si sites with ^1^H nuclei in their vicinity, originating either from Si-OH groups or from Si(nAl) units with adjacent H atoms.

**Figure 9:**
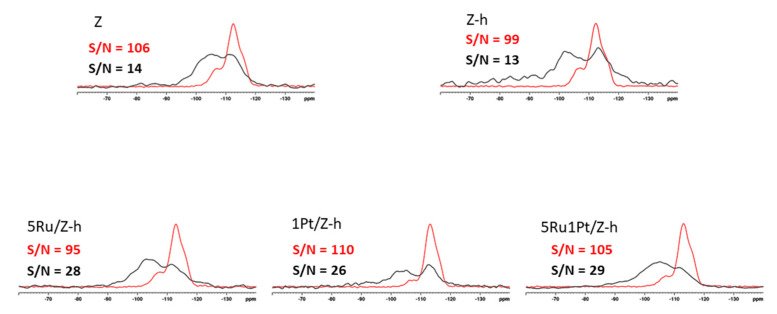
Comparison of the ^1^H→^29^Si CP (black) and single-pulse (red) ^29^Si NMR spectra of the studied zeolites.

Figure 9 shows the overplayed ^1^H→^29^Si CP–MAS spectra and the ^29^Si single-pulse spectra of some of the studied zeolite materials. The ^1^H→^29^Si CP spectra of all materials showed a low intensity resonance at approximately –92 ppm characteristic of Si(2OH) units, as well as a resonance at approximately –102 ppm indicating the presence of Si(1OH) groups. There was also an enhancement of the Si(0Al) resonances at –112 and –115 ppm, due to possible polarization transfer from neighboring silanol protons and/or coordinated water molecules. The resonance at –102 ppm was partially overlapped by the signal of Si(1Al) species at –107 ppm; therefore the actual Si/Al ratio might be slightly underestimated. The ^1^H→^29^Si CP–MAS technique is not quantitative since the effectiveness of magnetization transfer depends on Si–H internuclear distances and the relaxation rates and local dynamics of the structural fragments, which vary from one chemical environment to another. Considering the high number of scans necessary to achieve a reasonable signal-to-noise ratio (S/N) in the ^1^H→^29^Si CP spectra (NS~20,000) of all materials, we can conclude that the CP efficiency was very low and therefore we can assume that the quantity of the silanol groups was very low. For comparison, in the quantitative single pulse experiment, a much higher S/N of 110 was achieved with only 400 scans (Figure 9). The S/N ratio detected in the ^1^H→^29^Si CP spectra of ZSM-5 and Z-h was lower by a factor of 2 as compared to the S/N ratio in the spectra of the Ru-, Pt- and Ni-modified zeolites, obtained under identical experimental conditions (NS, relaxation delay, mixing time, etc.). Therefore we can qualitatively assume that the higher CP efficiency in the metal modified zeolites is due to the presence of a larger number of silanol functions in these materials.

### 2.2. Catalytic Activity for Phenol Preparation

**Scheme 1:**
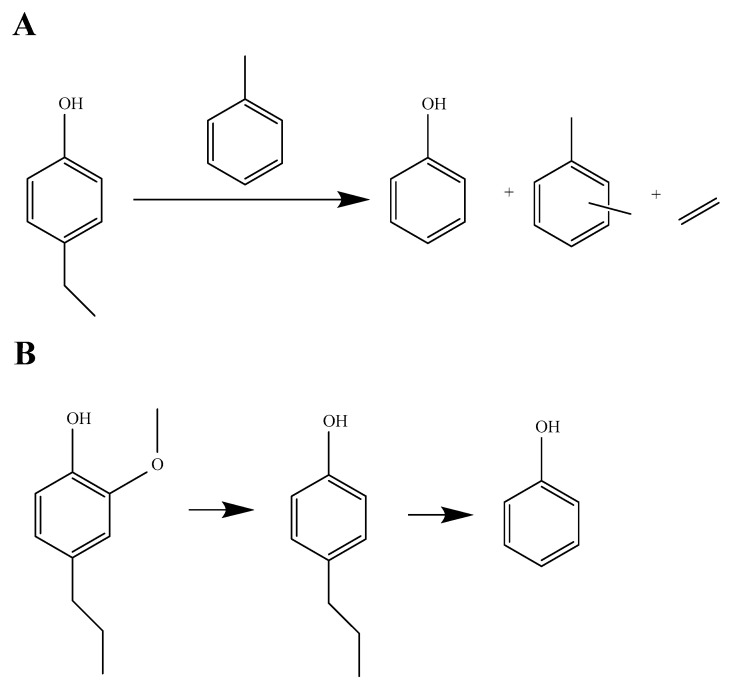
4-ethylphenol (**A**) and 2-methoxy-4-propylphenol (**B**) transformations.

The obtained hierarchical zeolite samples (Z-h) were studied and compared with the parent ZSM-5 in dealkylation of 4-ethylphenol and transalkylation of 4-ethylphenol with toluene (Scheme 1A, Table 3). The effect of the formed mesopores led to better diffusion of reactants and products, which was more pronounced at lower temperatures in dealkylation of 4-ethylphenol. The process of transalkylation of 4-ethylphenol with toluene was more significantly affected by the presence of mesopores in Z-h than the dealkylation of 4-ethylphenol, showing much higher catalytic activity than the initial ZSM-5.

Significant differences were registered in the stability of ZSM-5 and Z-h. The ZSM-5 catalyst showed a decrease in activity after three reaction cycles (Table 3) and the effect was more pronounced for transalkylation of 4-ethylphenol with toluene (65.4% versus 75.5%). By comparison, a negligible decrease in activity of the Z-h catalyst in transalkylation of 4-ethylphenol with toluene was registered (85.4% versus 86.5%).

The Ni-, Ru- and Pt-modified Z-h catalysts were studied in hydrodemethoxylation of 2-methoxy-4-propylphenol (Scheme 1B).

**Figure 10:**
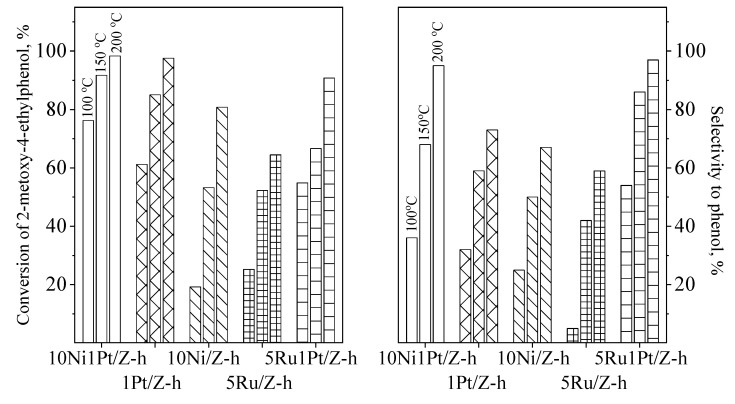
The catalytic activity (**left**) and selectivity to phenol(**right**) of the Ni-, Ru-, and Pt-modified hierarchical ZSM-5 zeolites in hydrodemethoxylation of 2-methoxy-4-propylphenol.

The conversion of 2-methoxy-4-propylphenol and the selectivity to phenol were compared at 100, 150 and 200 °C for all catalysts (Figure 10). Both conversion and phenol selectivity increased with temperature. The presence of acidic and metallic centres is important for the transformation of 2-methoxy-4-propylphenol to phenol.

The Pt-modified ZSM-5 sample showed much higher catalytic activity than that of the Ni- and Ru-containing ones. The Ru-containing catalyst (5Ru/Z-h) had the lowest activity in the hydrodeoxygenation of 2-methoxy-4-propylphenol. Among the three monometallic catalysts, 1Pt/Z-h possessed the highest selectivity to phenols at all temperatures, but did not reach 80% even at 200 °C. However, adding platinum to 10Ni/Z-h and 5Ru/Z-h created bimetallic catalysts (10Ni1Pt/Z-h, 5Ru1Pt/Z-5) with higher activity and significantly high selectivity to phenol. Only phenols were formed on 10Ni1Pt/Z-h and 5Ru1Pt/Z-5 at 200 °C. Both bimetallic catalysts selectively catalysed the hydrogenolysis of the C-O bond of the methoxy group to form 4-propylphenol, which was then dealkylated to phenol on the acid sites of the catalyst. The higher hydrogenolysis activity of the 10Ni1Pt/Z-h catalyst was probably due to the higher degree of reduction in NiO in the presence of platinum, as this matches findings for other Pt/Ni catalysts [41,42]. The simultaneous modification by nickel and platinum led to higher activity and selectivity to phenol. This effect could be explained by the stabilization of Al in tetrahedral position in 10Ni1Pt/Z-h as suggested by NMR in comparison to 1Pt/Z-h, and by the increase in the metal dispersion and reducibility due to the additive effect of Pt in 10Ni1Pt/Z-h.

**Figure 11:**
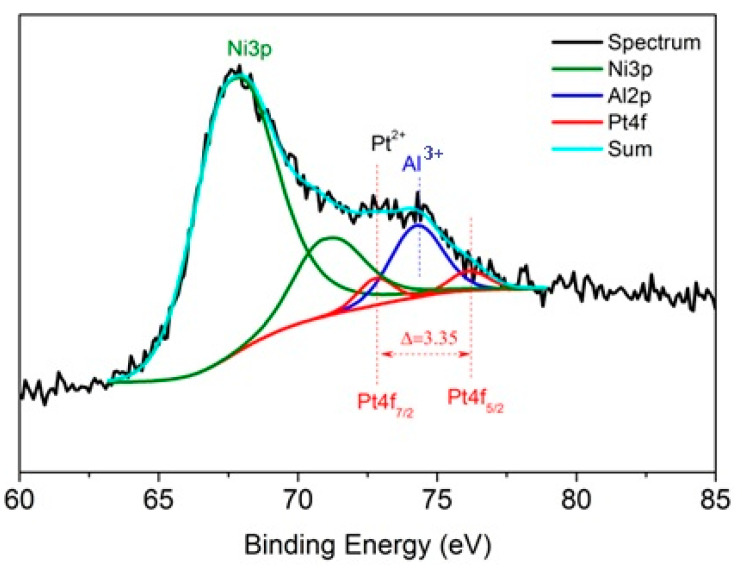
XPS spectra of 10Ni1Pt/Z-h zeolite.

We performed XPS analysis to characterize the surface sites in 10Ni1Pt/Z-h (Figure 11). The presence of peaks for Pt 4f_7/2_ with binding energy of 72.7 eV and Pt 4f_5/2_ binding energy of 76.1 eV was determined in the XPS spectra, which was due to the presence of Pt^2+^ ions. The simultaneous presence of Pt^0^ and Pt^2+^ was found in 10Ni1Pt/Z-h as indicated by TEM and XPS data. The peak for Ni 2p, binding energy in the range of 853–855 eV due to Ni in nickel oxide or nickel hydroxide, was registered as well. The nickel content was predominantly exposed to the zeolite surface and could participate in the reaction as active sites, whereas only a small part of the Pt^2+^ was accessible to the reactant molecules after its in situ reduction before the catalytic experiment.

The bimetallic 5Ru1Pt/Z sample showed lower catalytic activity in hydrodemethoxylation of 2-methoxy-4-propylphenol but higher selectivity to phenol in comparison to 5Ru/Z-h.

## 3. Experimental

### 3.1. Materials

The reagents used for the preparation of the ZSM-5 zeolite were sodium hydroxide (NaOH, analytical grade, Aldrich, Darmstadt, Germany), aluminum sulphate (Al_2_(SO_4_)_3_·18H_2_O, analytical grade, Aldrich), sodium carbonate (Na_2_CO_3_, analytical grade, Aldrich), tetraethyl orthosilicalite (TEOS, analytical grade, Aldrich), silicon(IV)oxide, 40% in H_2_O colloidal dispersion (SiO_2_, analytical grade, Aldrich), tetrapropylammonium hydroxide, 40% *w*/*w* aqueous solution (TPAOH, analytical grade, Aldrich) and H_2_SO_4_ (Aldrich). The chemicals used in the treatment were hydrofluoric acid (40% aqueous solution, Aldrich) and ammonium fluoride (Aldrich). All chemicals were used as received without any purification.

### 3.2. Synthesis of ZSM-5 Zeolite

The synthesis of ZSM-5 was carried out by hydrothermal seed-assisted synthesis with some modifications [25]. The molar ratio of the synthesis gel corresponded to 36.3 Na_2_O:3 Al_2_O_3_:100 SiO_2_:25.2 H_2_SO_4_:3000 H_2_O. NaAlO_2_ and NaOH were mixed and the colloidal silica was added to the solution under continuous stirring over several minutes. 1 wt% of the seed suspension was added to the gel mixture. The obtained gel was heated at 80 °C, after which the solution of sulphuric acid was slowly added to the gel and the mixture was stirred for 1 h. The obtained gel was transferred to autoclaves and hydrothermally synthesized at 180 °C for 24 h. Subsequently, the ZSM-5 powder was filtered, washed with water and dried at 100 °C overnight.

### 3.3. Preparation of Hierarchical ZSM-5

The initial ZSM-5 was treated with a mixture of hydrofluoric acid and ammonium fluoride. This mixture was prepared by adding 0.15 g HF (40%) to 60 mL water followed by 30 g NH_4_F. The mixture was stirred until a clear solution was formed. At this point, 500 mg ZSM-5 was added to the solution of HF and NH_4_F and stirred for 1 min, after which the sample was separated from the mixture by centrifugation. The sample was washed three times with distilled water. The parent sample was denoted as Z, and its hierarchical form as Z-h.

### 3.4. Modification with Ni, Ru or/and Pt Nanoparticles of Hierarchical ZSM-5 Zeolite

An impregnation technique with nickel, ruthenium and platinum salts was applied for loading of 10 wt%, 5 wt% and 1 wt% metals, respectively. The supports were heated at 160 °C for 2 h before the impregnation procedure.

A monometallic Ni-modified Z-h sample was prepared by the following procedure: 330 mg Ni(NO_3_)_2_·6H_2_O was dissolved in 10 mL ethanol and added to 300 mg Z-h support by stirring for 10 min. The sample was then dried at 80 °C for 18 h and calcined at 450 °C for 3 h at a rate of 1 °C/min. The sample was denoted as 10Ni/Z-h.

A monometallic Pt-modified Z-h sample was prepared by dissolving 40.4 mg Pt(II) acetyl acetonate in 40 mL ethanol and subsequently adding the solution to 2 g Z-h support by stirring at room temperature for 1 h. The ethanol solvent was removed in a rotary vacuum evaporator at 40 °C. The sample was then dried at 70 °C for 18 h and calcined at 450 °C for 3 h at a rate of 1 °C/min. The sample was denoted as 1Pt/Z-h.

A monometallic Ru-modified Z-h sample was prepared by dissolving 53.4 mg RuCl_3_ in 40 mL water and subsequently adding the solution to 500 mg Z-h support by stirring at room temperature for 12 h. The solvent was removed in a rotary vacuum evaporator at 40 °C. The sample was then dried at 110 °C for 12 h and calcined at 450 °C for 3 h at a rate of 1 °C/min. The sample was denoted as 5Ru/Z-h.

A bimetallic Pt- and Ni-modified Z-h sample was prepared by the following procedure: A solution of 334.95 mg Ni(NO_3_)_2_ × 6H_2_O in 4 mL ethanol was added to 609 mg 1Pt/Z-h sample and dried at 80 °C for 18 h. The precursor salt was decomposed in air at 450 °C at a rate of 1 °C/min for 3 h. The sample was denoted as 10Ni1Pt/Z-h.

A bimetallic Ru- and Pt-modified ZSM-5-h sample was prepared by the following procedure: A solution of 71.86 mg RuCl_3_ in 10 mL water was added to 609 mg 1Pt/Z-h sample and dried at 80 °C for 18 h. The precursor salt was decomposed in air at 450 °C at a rate of 1 °C/min for 3 h. The sample was denoted as 5Ru1Pt/Z-h.

### 3.5. Characterization

X-ray patterns were recorded by a Philips PW 1810/3710 diffractometer applying monochromatized CuK_α_ radiation (40 kV, 35 mA). Crystallite sizes of the metal oxides were determined by the Sherrer equation using FWHM values with full profile fitting methods. For the identification of crystalline phases, the ICDD database was used [26].

The specific surface area, pore volume and size of all samples were determined from N_2_ physisorption isotherms collected at −196 °C using an AUTOSORB iQ-C-MP-AG-AG (Quantachrome Instruments, Anton Paar brand, Boynton Beach, FL, USA). Samples were pretreated at 350 °C in vacuum.

The temperature-programmed reduction–thermogravimetric analysis (TPR–TGA) investigations were performed with a STA449F5 Jupiter-type instrument (NETZSCH Gerätebau GmbH, Selb, Germany) equipped with a SiC furnace and sensor holders TG/TG-DSC/TG-TDA type S, temperature range of RT to 1600 °C. In a typical measurement, 20 mg of a sample was placed in a microbalance ceramic crucible and heated in a flow of 5 vol% H_2_ in Ar (100 cm^3^/min) to 500 °C at 5 °C/min and a final hold-up of 1 h. Prior to the TPR experiments the samples were treated in situ in an air flow (10 °C/min) up to 500 °C, followed by a hold-up of 1 h.

The TEM images were taken on a JEOL JEM 2100 TEM (200 kV) (JEOL, Tokyo, Japan). The samples were suspended in a small amount of ethanol and a drop of the suspension was deposited onto a copper grid covered by carbon supporting film and dried at ambient temperature.

ATR–FTIR spectra were recorded by means of an IRAffinity-1 “Shimadzu” Fourier Transform Infrared (FTIR) spectrophotometer with MIRacle Attenuated Total Reflectance Attachment. The instrument was equipped with a temperature-controlled, high-sensitivity DLATGS detector and an ATR attachment with KRS-5 prism. In general, 50 scans and 4 cm^−1^ resolution were applied. The spectral data were processed with IR solution software.

NMR spectra were recorded on a Bruker Avance II+ 600 NMR spectrometer operating at 600.01 MHz ^1^H frequency (119.21 MHz for ^29^Si, 156.34 MHz for ^27^Al), using a 4 mm solid-state CP/MAS dual ^1^H/X probehead. The samples were loaded into 4 mm zirconia rotors and spun at magic-angle spinning (MAS) rates of 10 kHz for ^29^Si spectra and 14 kHz for ^27^Al spectra. The quantitative ^29^Si NMR spectra were recorded with a one-pulse sequence, 90° pulse length of 4.5 μs, 3 K time domain data points, spectrum width of 29 kHz, 400 scans and a relaxation delay of 120 s. The spectra were processed with an exponential window function (line broadening factor 10) and zero-filled to 16 K data points. ^27^Al spectra were recorded with a one-pulse sequence, 90° pulse length of 2.8 μs, 128 K time domain data points, spectrum width of 780 kHz, 1024 scans and a relaxation delay of 0.5 s. The spectra were processed with an exponential window function (line broadening factor 10). The ^1^H→^29^Si cross-polarization MAS (CP-MAS) spectra were acquired with the following experimental parameters: ^1^H excitation pulse of 3.6 μs, 2 ms contact time, 5 s relaxation delay, more than 20,000 scans, MAS rate of 10 kHz. A ^1^H SPINAL-64 decoupling scheme was used during CP acquisition experiments.

FTIR experiments were performed using a Nicolet Compact 640 spectrometer via the self-supported wafer technique with pyridine (Py) (7 mbar) as probe molecule. Self-supported pellets (10 × 20 mm) were pressed from the samples, placed into the IR cell, heated up to 400 °C in high vacuum (10^−6^ mbar) at a rate of 10 °C/min and dehydrated for 1 h. Following 30 min contact with Py at 200 °C, the sample was evacuated at 100, 200, 300 and 400 °C for 30 min. After each evacuation step a spectrum was recorded at the IR beam temperature with a resolution of 2 cm^−1^. The spectra were normalized to 5 mg/cm^2^ weight of the wafers for comparison.

The film composition and electronic structure were investigated by X-ray photoelectron spectroscopy (XPS). The measurements were carried out on an AXIS Supra electron spectrometer (Kratos Analitycal Ltd., a Shimadzu Group Company) with base vacuum in the analysis chamber of ~10^−7^ Pa. The spectra were recorded using achromatic AlKα radiation with photon energy of 1486.8 eV and a charge neutralisation system. The energy scale was calibrated by normalizing the C 1s line of adsorbed adventitious hydrocarbons to 284.8 eV. The binding energies (BE) were determined to an accuracy of ±0.1 eV. The deconvolutions of the peaks were performed using ESCApe^TM^ (Kratos Analytical Ltd., Manchester, UK) software.

The transition and noble metal content of the samples was determined by ICP-OES analysis. Samples were digested in order to make the components water-soluble. 50 mg of zeolite was mixed with 10 mL cc. HF in a polymer beaker and the formed SiF_4_ was evaporated in a water bath until totally dried. The HF treatment was repeated, and the remaining metal components were dissolved in 10 mL aqua regia. The acidic solvents were evaporated in the water bath and the dissolution procedure was repeated. The formed clear solution was transferred to a 50 mL volumetric flask and analyzed with a Spectro Genesis type simultaneous axial plasma ICP-OES spectrometer. For the determination of Ni content, a MU33 type multi-element standard solution was applied (CPAChem, M8A96.K1.5N.L5). Pt and Ru content was determined with the help of an 8 multi-element standard solution SPECTRASCAN SS-018313.

### 3.6. Catalytic Activity Measurements

Prior to the catalytic tests, samples were pretreated for 1 h in hydrogen (60 mL/min) up to 400 °C. Dealkylation, transalkylation and hydrodemethoxylation of 2-methoxy-4-propylphenol and 4-ethylphenol was studied at atmospheric pressure using a fixed-bed flow reactor with 30 mL/min carrier gas (hydrogen for hydrodemethoxylation and nitrogen for dealkylation and transalkylation). In the reaction 50 mg of the sample (particle size 0.2–0.8 mm) was tested and diluted with 50 mg glass beads of the same diameter, previously checked to be inactive. The reactor itself was a quartz tube of 15 mm inner diameter, with the catalyst bed at the middle. A thermocouple was positioned in the catalyst bed for accurate measurement of the catalyst temperature. All gas lines of the apparatus were heated continuously at 383 K in order to minimize reactant and products adsorption on the tube walls. The gas stream passed through a saturator filled with 4-ethylphenol, toluene and 2-methoxy-4-propylphenol equilibrated at 80 °C. The reactant was fed into the reactor at a flow rate of 30 mL/min and catalytic tests were carried out in the temperature range 150–300 °C depending on the reactants used. The reaction steady state was established after 30 min in each temperature. On-line analysis of the reaction products was performed using a NEXIS GC-2030 ATF with a 30 m HP 5MS capillary column.

## 4. Conclusions

It was found that hierarchical ZSM-5 zeolite is a suitable support for the preparation of mono- and bimetallic Ni-, Ru- and Pt-containing catalysts with high activity in transformation of 2-methoxy-4-ethylphenol and 4-ethylphenol to phenol. Formation of finely dispersed nickel, ruthenium and platinum particles was registered on the catalysts. Among the studied catalysts, 10Ni1Pt/Z-h showed the highest activity and selectivity for phenol production. The 5Ru1Pt/Z-h bimetallic catalyst showed higher selectivity but lower activity in hydrodemethoxylation of 2-methoxy-4-propylphenol. FTIR spectroscopic investigation of adsorbed pyridine evidenced that the acidity of the catalysts was preserved by hierarchization, i.e., by treatment with a HF and NH_4_F mixture. It was also found in Ni-containing catalysts that a portion of the nickel was incorporated into the zeolite lattice to ionic positions due to the applied impregnation method. The additive effect of Pt was related to the increase in the metal dispersion and enhancement of NiO reducibility. Bimetallic 10Ni1Pt-modified hierarchical ZSM-5 zeolite is a highly active and stable catalyst for selective production of biomass-derived phenol via gas-phase hydrodeoxygenation of 2-methoxy-4-propylphenol.

## Figures and Tables

**Table 1:** Physico-chemical properties of the Z, Z-h and Ni-, Ru- and Pt-modified Z-h zeolites.

Samples	BET Surface Area, (m^2^/g)	Pore Volume, (cm^3^/g)	Mesopore Volume, (cm^3^/g)	Crystallite Size, (nm)	Reducibility ^a^, (%)
Z	525	0.35	0.05	-	-
Z-h	410	0.27	0.17	-	-
10Ni/Z-h	351	0.26	0.18	8 (NiO)	80 (NiO)
10Ni1Pt/Z-h	335	0.32	0.19	8 (NiO)	100 (NiO)
1Pt/Z-h	404	0.32	0.20	-	100
5Ru/Z-h	330	0.33	0.16	23 (RuO_2_)	60 (RuO_2_)
5Ru1Pt/Z-h	282	0.26	0.15	35 (RuO_2_)	77 (RuO_2_)

^a^ Total weight loss related to the calculated theoretical weight loss for the reduction of the corresponding oxide to its metallic state.

**Table 2:** Area of the signals of the different Si and Al species, obtained by deconvolution of the spectral patterns in the ^29^Si and ^27^Al spectra.

Samples	Q^4^(1Al) −107 ppm	Q^4^(0Al) −113 ppm	Q^4^(0Al) −115 ppm	Si/Al	FAl 62–58 ppm %	FAl distorted 40–38 ppm, %	EFAl 7–0 ppm %
Z	17	64	19	24	91	-/-	9
Z-h	19	65	16	21	72	-/-	18
5Ru/Z-h	20	60	20	20	80	11	9
10Ni/Z-h	13 + 7	49	31	20	100	-/-	-/-
1Pt/Z-h	3 + 8	75	14	36	53	20	5 + 13 + 9 *
5Ru1Pt/Z-h	10	68	22	40	41	50	1 + 8
10Ni1Pt/Z-h	14	71	15	29	32	62	4 + 3*

* A signal at −11 ppm, of unknown origin.

**Table 3:** Catalytic activity of the initial and hierarchical ZSM-5 zeolite in dealkylation of 4-ethylphenol and transalkylation of 4-ethylphenol with toluene.

Samples	4-Ethylphenol Dealkylation, Conversion, %			Transalkylation of 4-Ethylphenol with Toluene at 300 °C		
	170 °C	200 °C	250 °C	Conversion, %	Selectivity to Phenol, %	Selectivity to Xylenes, %
Z	11.6	84.0	87.7 (85.7) *	75.5 (65.4) *	54.7 (53.6) *	45.3 (46.4) *
Z-h	38.2	87.4	90.1 (89.8) *	86.5 (85.4) *	35.4 (34.8) *	64.6 (65.2) *

* Results were obtained after the 3rd reaction cycle.

## Supplementary Materials

The following are available online, Figure S1: Linear fit of p/[n(p0−p)] against p/p0 plots, Figure S2: SAED of 10Ni1Pt/Z-h, Table S1: Chemical composition of the studied samples.

## Sample Availability

Samples of the hierarchical ZSM-5 zeolites are available from the authors.
