# Supplementary material for: Selective Production of Phenol on Bifunctional, Hierarchical ZSM-5 Zeolites
Source: Molecules. 2021 Jun 11;26(12):3576. doi: 10.3390/molecules26123576 (PMC8231192; doi:10.3390/molecules26123576)
Supplement: Supplementary file 1 [file molecules-26-03576-s001.zip › molecules-1240229-supplementary.pdf]

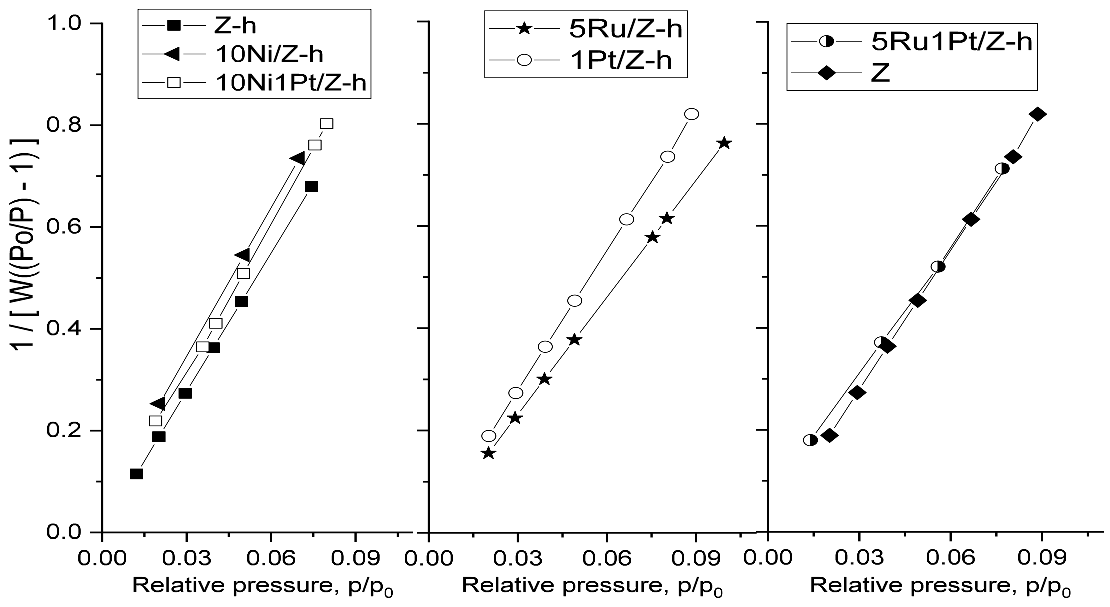

Figure S1. Linear fit of p/[n(p_0-p)] against p/p_0 plots

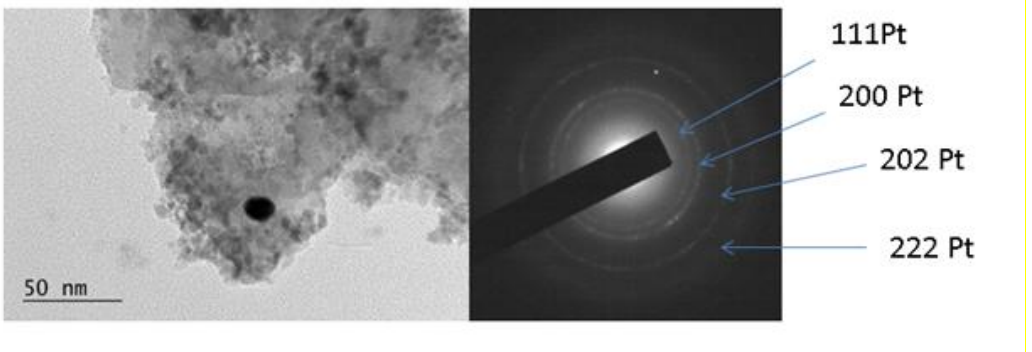

Figure S2. SAED of 10Ni1Pt/Z-h (B) (Phases identified: Pt cubic, a=3.87800, #96-101-1115 and Ni hexagonal, a= 2.62200 A c= 4.32100 A, # 96-901-3008)

Table S1. Chemical composition of the studied samples

Samples	Content of Ni, wt. %	Content of Pt, wt. %	Content of Ru, wt. %
10Ni/Z-h	9.86	-	-
10Ni1Pt/Z-h	9.92	0.98	-
Pt/Z-h	1.02	-	-
5Ru/Z-h	-	-	4.83
5Ru1Pt/Z-h	-	0.92	4.89
